# Transition Behaviors of Configurations of Colloidal Particles at a Curved Oil-Water Interface

**DOI:** 10.3390/ma9030138

**Published:** 2016-02-26

**Authors:** Mina Lee, Ming Xia, Bum Jun Park

**Affiliations:** Department of Chemical Engineering, Kyung Hee University, Yongin, Gyeonggi-do 17104, Korea; mina.lee@khu.ac.kr (M.L.); mxia19861123@gmail.com (M.X.)

**Keywords:** colloids, fluid-fluid interfaces, assemblies, interactions, heterogeneity, self-potentials

## Abstract

We studied the transition behaviors of colloidal arrangements confined at a centro-symmetrically curved oil-water interface. We found that assemblies composed of several colloidal particles at the curved interface exhibit at least two unique patterns that can be attributed to two factors: heterogeneity of single-colloid self-potential and assembly kinetics. The presence of the two assembly structures indicates that an essential energy barrier between the two structures exists and that one of the structures is kinetically stable. This energy barrier can be overcome via external stimuli (e.g., convection and an optical force), leading to dynamic transitions of the assembly patterns.

## 1. Introduction

Colloidal particles strongly and irreversibly attach to fluid-fluid interfaces [1,2,3]. This adsorption leads to a reduction in the interfacial tension between the immiscible fluid phases, thereby causing thermodynamic or kinetic stabilization of the interface [1,3,4]. Interactions between interface-trapped particles are abnormally strong and long-ranged, which differs significantly from DLVO (Derjaguin-Landau-Verwey-Overbeek) interactions where identical particles are dispersed in a single fluid phase, such as water [5,6,7,8,9,10,11,12,13,14,15,16,17,18]. In addition to such interparticle interactions, assembly/microstructures and rheological properties of colloidal particles confined at fluid-fluid interfaces have been extensively investigated over the last three decades; this is because such particle-laden interface systems can be used for various applications, such as solid surfactants (e.g., Pickering emulsions), material transfer/delivery, and microemulsion catalytic reactors [19,20,21,22,23,24,25,26,27,28,29].

Control over the assembly of colloidal particles with versatile functionalities at fluid-fluid interfaces can be used to establish micro- and nano-scale building blocks [2]. Consequently, the study of small-scale measurements (e.g., pair interactions, assemblies, and micromechanics) can provide a direct link to measurements (e.g., micro- and macro-structures and rheology) on a larger scale for materials that are composed of the same types of constituents, thereby potentially offering fundamental ideas and design rules for the construction of hierarchical structures and materials with specific properties. For example, two particle interactions with a few microns in size at a planar oil-water interface were measured via optical laser tweezers, and the magnitude of the obtained interaction forces was found to depend on the particle pairs (*i.e.*, the pair interaction heterogeneity) [29]. Based on Monte Carlo (MC) simulations and experimental observations, it was found that the measured interaction heterogeneity of the particles directly affects the conformation of the equilibrium microstructure of two-dimensional colloidal suspensions consisting of identical particles. Similarly, it was reported that the interactions between polystyrene particles with a few hundred micrometers in diameter at a centro-symmetrically curved oil-water interface are repulsive, which can affect the formation of diverse assembly patterns composed of several particles [30]. The diversity in the assembly structures can be attributed to heterogeneity in interparticle interactions. However, it should be noted that the magnitude of the interaction potentials for each interacting particle, rather than the pair interaction magnitude, is heterogeneous, and thus, the heterogeneity of the potentials of individual particles likely affects their assembly behaviors.

The heterogeneous properties in colloidal interaction systems are crucial because the assumption of particle interaction homogeneity can result in a deviation from predicted large-scale properties for systems composed of identical particles. Such variations could occur in the structural and rheological properties of a suspension. To understand the heterogeneity of colloidal systems on a single particle level, a new concept of self-potentials possessed by individual particles trapped at an oil-water interface has been developed [31]. To characterize the self-potentials of individual colloidal particles, energy minimization is performed numerically when the interface-trapped particles form uniquely arranged structures. Notably, it was demonstrated that the self-potentials represent the dipole strength of individual particles at the interface, which can account for electrostatic dipolar interactions with abnormally strong and long-ranged properties [5,6,7,8,9].

In this work, we focus on studying transition behaviors of assembly configurations composed of several particles at a curved oil-water interface, based on the self-potential model. We experimentally observed that configurations of polystyrene particles at the curved interface that are repulsive to each other can be varied between two different structures by introducing external stimuli (convection and optical forces). This implies that one of the structures is kinetically stable and the other is thermodynamically stable. We believe that these structural transitions of the particle assemblies are due to the effects of particle interaction heterogeneity and assembly kinetics. We use MC simulations to systematically understand the role of the two factors, employing the heterogeneity of the self-potentials. This paper is organized as follows. First, we present experimental observations of the assembly pattern formation and the structural transition induced by convective flow or laser irradiation. Then, we present data from MC simulations to quantitatively investigate the effect of the interaction heterogeneity and assembly kinetics on the assembly behaviors. Finally, we describe the detailed experimental procedure for the preparation of particles and the formation of assembly patterns at the curved oil-water interface. This is followed by a description of the simulation method.

## 2. Results and Discussion

### 2.1. Experimental Observations of Assembly Configurations

**Figure 1:**
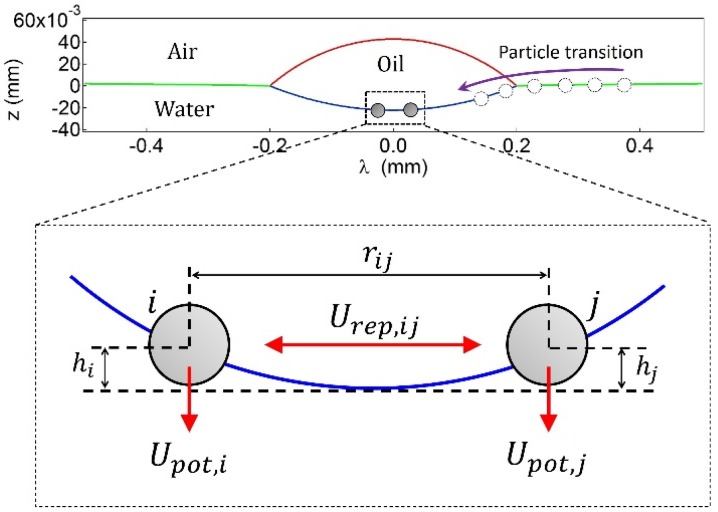
Schematics for particle interactions at the curved oil-water interface.

A convex oil lens was formed by gently placing a small amount of *n*-decane on the surface of ultrapure water in a Petri dish (Figure 1). Polystyrene particles with 200 μm in diameter (2*R*) were individually inserted to a curved decane-water interface in which the curvature of the interface can be determined by solving the nonlinear Yong-Laplace equation [23,32]. The particles trapped at the interface tend to collect near the bottom of the oil lens due to gravity and also experience electrostatic repulsions [5,6,7,8,9], forming unique assembly structures. We refer the readers to the Section 3 for the detailed experimental methods.

It was observed that the assembly composed of eight to ten particles at the curved oil-water interface shows two unique patterns depending on the number of particles. The assembly of eight particles adopts either the configuration with one inner particle surrounded by seven outer particles (1@7) or the structure with two inner particles surrounded by six outer particles (2@6). Nine and ten particle assemblies show either two inside structures (2@7 and 2@8) or three inside structures (3@6 and 3@7), respectively. We believe that these assembly behaviors can be attributed to heterogeneity of self-potentials. The role of the self-potential heterogeneity that is incorporated with MC simulations is investigated in the next section.

**Figure 2:**
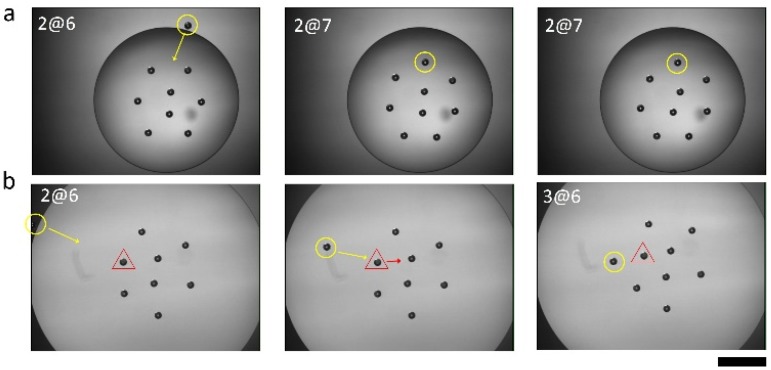
Experimental snapshots of the structure transitions when a ninth particle is added to the eight particle assembly: (**a**) from 2@6 to 2@7; and (**b**) from 2@6 to 3@6; the scale bar is 1mm.

The assembly behaviors also likely depend on a kinetic factor. A particle is added to the 2@6 assembly to experimentally observe the effect of assembly kinetics on the particle configurations. As shown in Figure 2a, as the added particle approaches along the direction of the line joining the two inner particles, it joins the outer particles forming the 2@7 structure. In contrast, when the inserted particle approaches the 2@6 structure at the angle of the line joining the two inner particles (Figure 2b), the nine particles consequently adopt the 3@6 structure. These experimental results indicate that the kinetic factor plays an important role in the formation of assembly structures.

**Figure 3:**
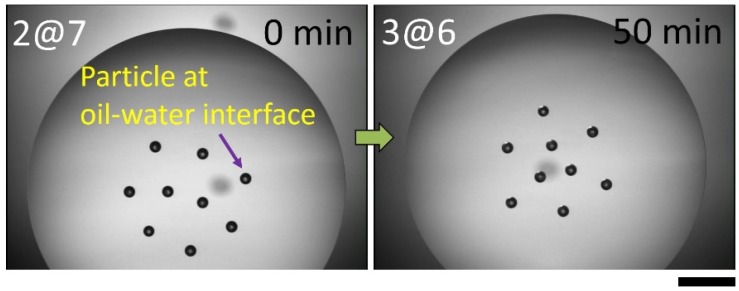
Structural transition form 2@7 to 3@6 assembly patterns at the curved oil-water upon oil drying; the scale bar is 1mm.

Interestingly, the assembly pattern can be transferred to a different structure via an external stimulus, such as convection due to oil drying. The sample cell containing the particles attached to the curved oil-water interface is uncovered under ambient conditions, allowing the oil to dry, enabling convection to occur. As shown in Figure 3, the assembly pattern consisting of nine particles at the interface initially shows two inner particles surrounded by seven outer particles (2@7). Upon oil drying, one of the outer particles enters inward to form an inner triangle enclosed by six outer particles (3@6) after a period of time. Since this structural change seldom occurs when the sample cell is covered and convection is suppressed, we believe that convection caused by oil drying leads to such a structural transition.

**Figure 4:**
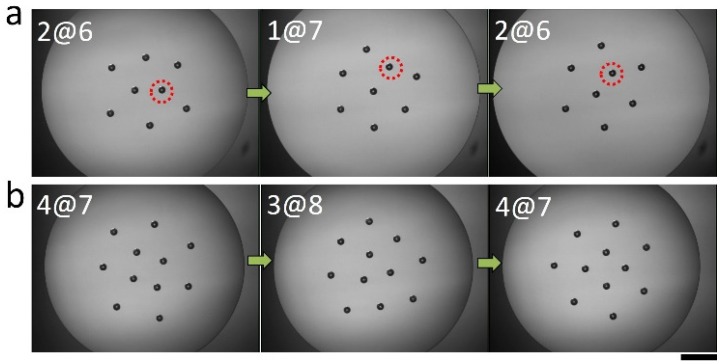
Transition of the assembly pattern activated by an optical force: (**a**) between 2@6 and 1@7; and (**b**) between 4@7 and 3@8; the scale bar is 1mm.

We also observed that the assembly configurations consisting of eight and eleven particles can also be altered by stimulating the structure via an optical force. Note that eleven particles also form two distinct patterns, either 3@8 or 4@7 structures. A laser beam (power ≈ 1 mW, wavelength = 650 nm) is introduced from a side port of the microscope, which reaches the focal plane through the aperture of a microscope objective. The rays of the laser beam are transmitted and reflected at the particle-fluid interface, as well as the fluid-fluid interfaces, generating an optical force that can randomly disturb the assembly structure [33]. Note that the sample cell is covered to minimize the effect of convection due to evaporating oil. As shown in Figure 4a, the assembly composed of eight particles initially exhibits a 2@6 structure. After approximately 15 min of irradiation with the laser beam, one of the inner particles (indicated by a red circle) moves outward to form the 1@7 structure. This configuration is maintained for a while, even after the removal of the laser beam. Further irradiation for another 15 min leads to the recovery of the original structure. Similarly, when eleven particles initially form the structure in which four particles are surrounded by seven outer particles (4@7), laser irradiation for ~15 min leads to a transition into the 3@8 structure, as shown in Figure 4b. The reverse transition (back to the original configuration), occurs upon exposure to the laser beam for another 15 min. In short, these observed experimental results likely demonstrate the presence of an energy barrier between the two assembly structures that can be overcome by an external stimulus, and one of the assembly structures should be kinetically stable. Next, we use MC simulations to systematically understand these assembly behaviors.

### 2.2. Assembly Behaviors via MC Simulations

There are two factors that likely determine the assembly structures: interaction heterogeneity and assembly kinetics. To understand the effects of interaction heterogeneity, we run MC simulations by introducing the heterogeneity of self-potentials that were previously measured [31].

**Figure 5:**
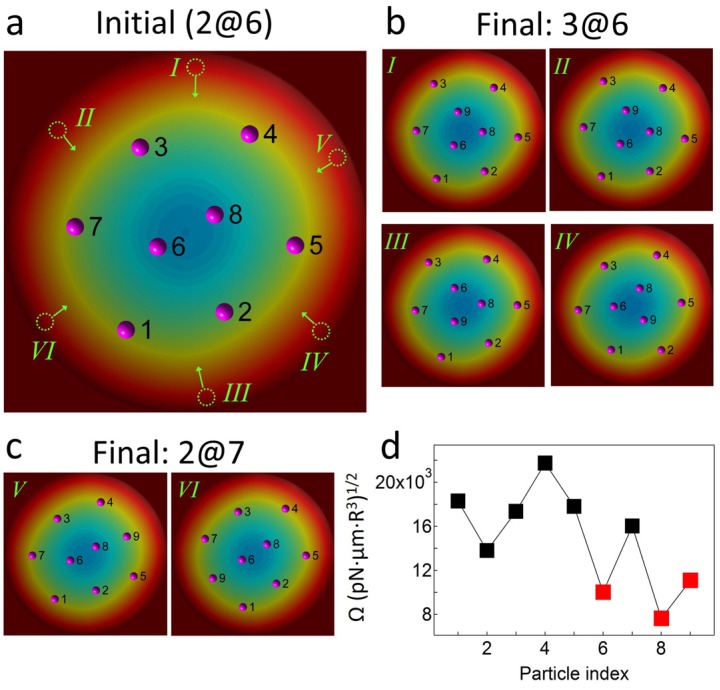
Path-dependent structural transition behavior. (**a**) The initial configuration of the 2@6 structure for the MC simulations. Particle 9 approaches the 2@6 structure form six locations, as indicated by the dotted circles (I-VI); (**b,c**) The configurations resulting from each run of the MC simulations; and (**d**) The self-potentials (Ω_i_) for the nine particles used in the MC simulations.

When self-potentials for the nine particles are randomly allocated for each run of the simulation, as described earlier, the particles form the 3@6 structure 34% of the time (68 out of 200 runs) and the 2@7 structure 66% of the time. To perform a more quantitative analysis, we select an arbitrary set of self-potentials for the nine particles, as shown in Figure 5d. The initial positions of the nine particles at the curved oil-water interface in the simulations are also randomly generated. Multiple runs of simulations show that the nine particles form the 3@6 structure 62% of the time (124 out of 200 runs). Among the simulation results that form the 3@6 structure, the particles with the three lowest self-potentials (particles 6, 8, and 9) almost always collect inward (~90% of the time). It is likely that the particles with lower self-potentials tend to possess the larger number of nearest neighbors and, as a result, collect inside of the structure. Among the remaining simulation results that assemble into the 2@7 structure (76 out of 200), particles 6 and 8, which possess the two lowest self-potentials, form the two inner particles 32% of the time. Additionally, at least one particle among the six or eight is always located inside the 2@7 pattern. Notably, when a homogeneous interparticle interaction is introduced to the simulations, the nine particles only form the 3@6 structure (100 out of 100). These simulation results demonstrate the importance of interaction heterogeneity in assembly behavior.

The assembly kinetics also significantly affects the formation of assembly patterns. We investigate the effect of the path of a particle that is added to the 2@6 assembly structure using MC simulations. We arbitrarily select a 2@6 configuration as the initial state for the simulations (Figure 5a) in which the self-potential values for the eight particles in Figure 5d are randomly selected among the regenerated self-potential values based on the gamma distribution, as described in the Section 3. Then, particle 9, which possesses the third-lowest self-potential in Figure 5d, is inserted into the equilibrium 2@6 structure from six locations (I-VI), as indicated by the dotted circles in Figure 5a. Note that this simulation geometry is analogous to the experimental conditions in Figure 2. As shown in Figure 5c, the 2@7 structure is obtained when the inserted particle approaches along the path that is aligned with the line joining the two inner particles, particles 6 and 8 (cases V and VI in Figure 5c). In the other cases, where particle 9 approaches the 2@6 structure at the angle of the line joining the two inner particles, the nine particles consequently form the 3@6 structure (cases I-IV in Figure 5b). Note that when forming the 3@6 structure, the indexes of the inner particles are always 6, 8, and 9, which possess the three lowest self-potentials.

**Figure 6:**
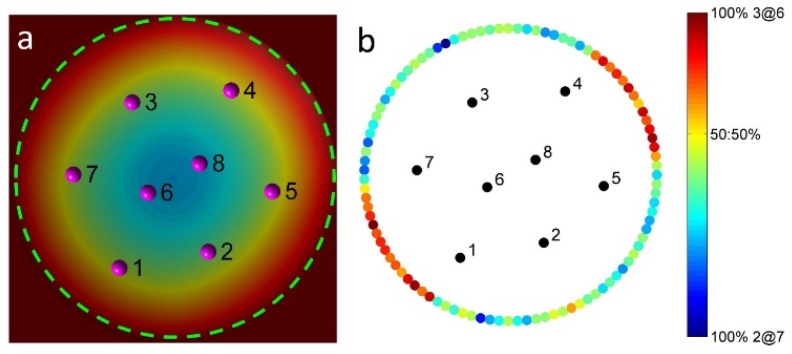
The probability of the formation of either the 2@7 or 3@6 structures upon the addition of particle 9 to the 2@6 structure. (**a**) Initial 2@7 configuration. The dashed circle represents the initial position of the inserted particle; (**b**) The probability of the two resulting structures is indicated by various colors on the initial position.

Further simulations (500 runs) are performed by adding particle 9 to the 2@6 structure, in which the initial position of particle 9 is arbitrarily located on the circular boundary, as indicated by the dashed circle in Figure 6a. The probability of the two resulting assembly patterns forming is indicated by various colors on the initial positions of the circular boundary in Figure 6b. Consistently, a higher probability (red) for the 3@6 formation is obtained when particle 9 approaches from the approximate directions of either 1-2 or 7-8 o’clock; for the other cases, the 2@7 assembly is preferred. Note that the probability (colors on the circular boundary in Figure 6b) of forming two unique structures (2@7 and 3@6) is not symmetric with respect to the line joining the two inner particles, particles 6 and 8, due to the heterogeneity of the self-potentials of the nine particles (Figure 5d). Interestingly, when one particle is added to the 2@6 configuration, the corresponding probabilities for the 3@6 and 2@7 structures to form are 71% and 29%, respectively. These differ from the probabilities in the case where the random initial configurations and the same self-potentials are used in the simulations (62% for 3@6 and 38% for 2@7). These simulation results, which depend on the initial configurations as well as the path of an inserted particle, demonstrate the effect of kinetics on determining the assembly patterns.

**Figure 7:**
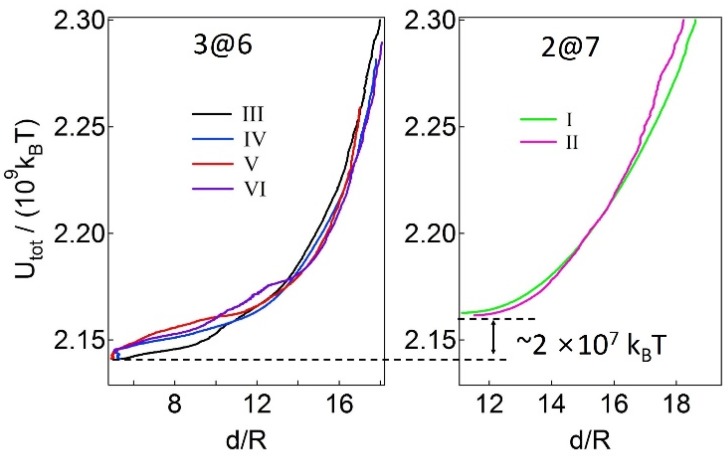
The total interaction energy (*U_tot_*) as a function of the radial distance, *d*, of particle 9 from the center of the oil lens while rearrangements occur.

The total interaction potential for the formation of the 3@6 and the 2@7 structures continuously decreases until particle 9 finds a position at which a local energy minimum likely exists, as shown in Figure 7. The magnitude of the total interaction potential for the 3@6 formation is ~2 × 10^7^ k_B_T lower than that of the 2@7 formation, suggesting that the 2@7 formation is a kinetically stable configuration. Similar results are obtained when either particle 6 or 8 is inserted into the 2@6 assembly (instead of particle 9) in the simulations.

**Figure 8:**
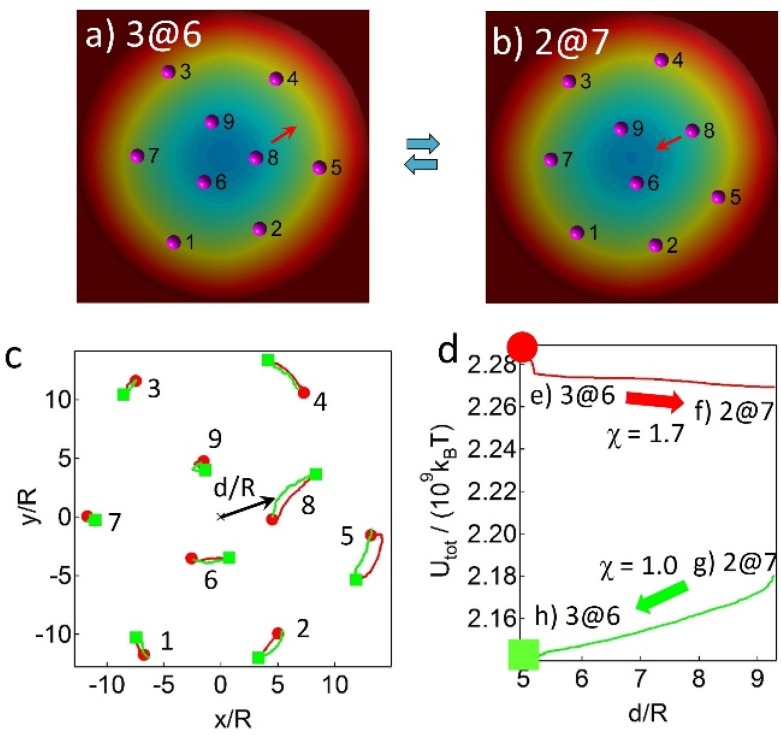

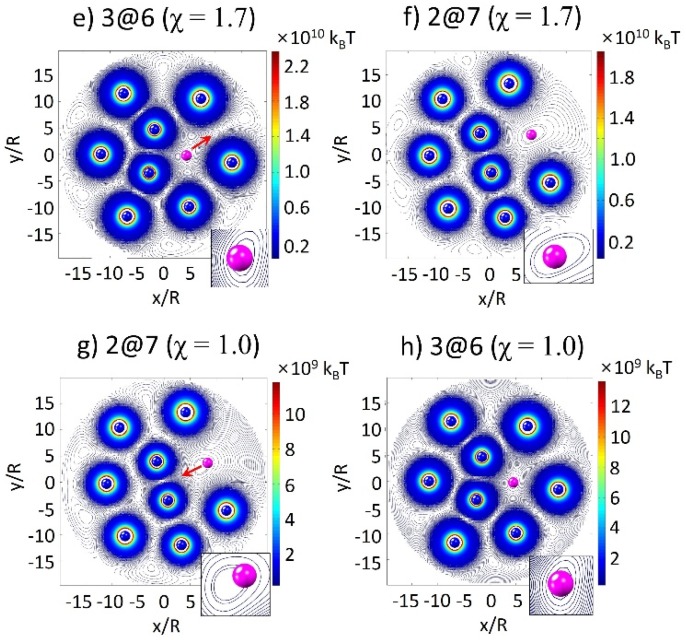
Magnitude of the critical self-potentials at which the structural transition occurs between the 3@6 and 2@7 patterns in MC simulations. The self-potential values in Figure 5d are used in the simulations. (**a**,**b**) The structural transition when the critical self-potential of particle 8, Ω_8_' = 1.70 × Ω_8_ ≈ 1.30 × 10^4^ (pNμmR^3^)^1/2^, is used in the simulation; (**c**,**d**) The corresponding particles’ trajectories; (**c**) and the total interaction energies; (**d**) when the transition between the 3@6 and 2@7 structures occurs; (**e**–**h**) The transition energy field (contour lines) at the moment of the structural transition with respect to particle 8. The pink dot indicates the position of particle 8 and the blue dots are the other eight particles. The contour lines near each blue particle is not shown because the potential is extremely high.

To understand the effects of the self-potential magnitude on the assembly configurations, we determine the critical value of the self-potentials to be the point for which the assembly structure transitions to a different structure. The three inner particles of the 3@6 structure typically possess the three lowest self-potentials, as described earlier, implying that a critical value of the self-potential of the inner particles should exist. To find this critical magnitude, the self-potential of one of the three inner particles (Figure 5d) is amplified in the MC simulations until the particle migrates outward, leading to the formation of the 2@7 structure (Figure 8a,b). It is found that particle 8 transfers to the outside when its self-potential is scaled by a factor of up to χ = 1.7, such that Ω_8_' = 1.70 × Ω_8_ ≈ 1.30 × 10^4^ (pNμmR^3^)^1/2^. At the moment of amplifying the self-potential of particle 8, the transient energy field with respect to particle 8 (Figure 8e) shows that the location of particle 8 in the 3@6 structure deviates slightly from the local energy minimum (inset in Figure 8e). This, consequently, displaces the particle in an outward direction to decrease the total energy until the particle reaches the position of the local energy minimum in the 2@7 structure (Figure 8f). Similarly, the reverse transition occurs when the amplified factor of particle 8 is removed (χ = 1.0), such that Ω_8_' = Ω_8_. As shown in the transient energy field (Figure 8g), particle 8 in the 2@7 structure moves inward due to deviations in the particle position caused by the transient local energy minimum. Eventually, particle 8 joins the two inner particles 6 and 9, and assembles into the 3@6 pattern (Figure 8h). The particles’ trajectories and the total interaction energies during the structural transitions are shown in Figure 8c,d, respectively. The solid symbols in Figure 8c indicate the initial positions of the particles before each transition occurs. Similarly, the critical self-potentials for particles 6 and 9 are found to be Ω_6_' = 1.56 × Ω_6_ ≈ 1.56 × 10^4^ (pNμmR^3^)^1/2^ and Ω_9_' = 1.17 × Ω_9_ ≈ 1.30 × 10^4^ (pNμmR^3^)^1/2^, respectively. The simulation results indicate that the transition tends to occur when the critical self-potentials are comparable to the value of the fourth lowest self-potential (the value of particle 2). In addition, upon replacing the value of Ω_8_ with the critical value of Ω_8_' = 1.30 × 10^4^ (pNμmR^3^)^1/2^, we run MC simulations with random initial configurations. The multiple runs of simulations show that the probability of forming the 3@6 structure dramatically decreases (7% of the time), compared to the case of the simulations when the original values of self-potentials in Figure 5d are used (62%). The increase in the self-potential value for particle 8 leads to the formation of the preferred 2@7 structure. In short, the simulation results demonstrate that the assembly structures depend on the relative strength of the self-potentials and the relative positions of the surrounding inner and outer particles.

**Figure 9:**
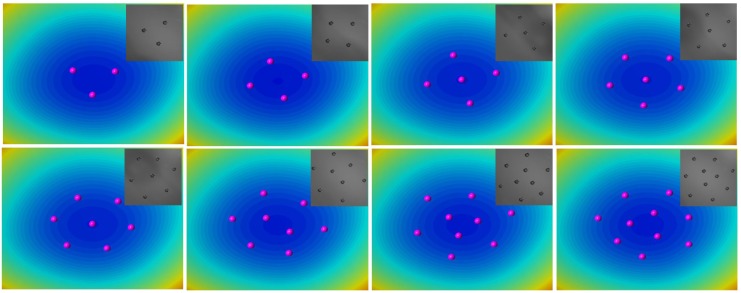
Examples of assembly patterns obtained by simulations and experiments when particles are added to the curved oil-water interface in a sequential manner.

Finally, it is likely that the pattern formation depends on the history of sequential addition of particles that belongs to the effect of assembly kinetics. One by one addition of particles to the curved interface, for example, results in assembly patterns of triangle, quadrangle, 1@4, 1@5, 1@6, 2@6, 3@6, and 3@7, as shown in the experimental and simulation results in Figure 9. The similar pattern formation, however, can also be observed when the same number of particles are simultaneously added to the interface. Therefore, we believe that it is not trivial to solely extract the role of the sequential addition because the two effects (*i.e.*, interaction heterogeneity and assembly kinetics) simultaneously affect the assembly behaviors of particles at the curved interface.

## 3. Materials and Methods

### 3.1. Preparation of Particles

Polystyrene (PS) particles that are 200 μm in diameter (2*R*) were prepared using a microfluidic device with co-flow geometry [23]. 30 wt% polystyrene (M.W. = 190 K, Sigma-Aldrich, Yongin, Korea) in methylene chloride (Sigma-Aldrich, Yongin, Korea) forms droplets out of a tapered inner glass capillary tube, and the outer continuous phase is water containing 2 wt.% polyvinyl alcohol (PVA, Sigma-Aldrich, Yongin, Korea), which prevents coalescence of the droplets. The generated droplets were collected in a pure aqueous solution and dried at an ambient temperature for several days. This particle dispersion was washed with pure water several times to remove excess PVA. To measure the surface potential of the PS particles, 30 wt.% PS dissolved in methylene chloride was dispersed in 2 wt.% PVA using an ultrasonic cleaner (Fisher Scientific, Seoul, Korea). The methylene chloride in the dispersed phase was removed at an ambient temperature for several days and the resulting particle dispersion was washed with pure water several times. The ζ-potential was then measured at approximately −20 mV (Beckman Coulter Delsa Nano-C, Indianapolis, IN, USA) due to the presence of PVA moieties on the particle surface that can consequently lead to repulsive interparticle interactions at an oil-water interface [23].

### 3.2. Formation of Convex Oil Lens

To generate a centro-symmetrically-curved oil-water interface, a small amount of *n*-decane is placed on the surface of water (resistivity > 18.2 MΩ·cm) in a Petri dish (Falcon, Yongin, Korea). A convex oil lens is formed and its shape (*i.e.*, air-water, air-oil, and oil-water interfaces in Figure 1) can be determined as a function of the radial distance, *λ*, via the Mathematica Player file provided by Burton *et al.*, [32] in which the parameter values are the surface tensions of the air-oil (*γ_ao_* ≈ 23.8 mN/m) and air-water (*γ_aw_* ≈ 72 mN/m) interfaces, the spreading coefficient (S_decane_ = −3.72 ± 0.24), and the density of decane (*ρ* = 730 kg/m^3^).

### 3.3. Particle Adsorption to the Curved Oil-Water Interface

Particles were individually inserted into the air-water interface outside the oil lens using a micropipette. The transition of a particle from the air-water interface to the centro-symmetrically curved oil-water interface occurs spontaneously, minimizing the attachment energy of the particle to the fluid interfaces (air-water, air-oil, and oil-water), as well as the potential energy due to gravity (Figure 1) [23]. To place several particles at the curved oil-water interface, a number of particles are initially spread at the planar air-water interface and then a small amount of oil is added onto the water surface, allowing the particles to transition to the curved oil-water interface. After introducing the particles, the sample cell is covered to minimize convection, if necessary. To observe a convection-induced structural change in the particles, the sample cell is uncovered, leading to evaporation of the oil. Note that since the bond number (the ratio of the gravitational force to the interfacial tension force) is sufficiently small (Bo ≈ 10^−3^), the local interface deformation around the particle is negligible. Microscopic snapshots are captured by a charge-coupled device (CCD) camera (Hitachi KP-M1AN, Daejeon, Korea) in order to image the assembly configurations. The obtained images are analyzed with ImageJ software [34].

### 3.4. Monte Carlo Simulation

We use Monte Carlo (MC) simulations to quantitatively analyze the assembly behaviors at the curved oil-water interface. *N* particles at the interface repel each other due to electrostatic repulsion, and the corresponding pair interaction between particles *i* and *j* is given by:

(1) $$\frac{U_{rep,ij}}{k_{B}T}=\frac{\Omega_{i}\Omega_{j}}{r_{ij}^{3}}=\frac{a_{ij}}{r_{ij}^{3}}$$

where *r_ij_* is the particle separation; k_B_ is the Boltzmann’s constant; and *T* is the temperature [6,18,30]. It has been reported that the electrostatic repulsive interactions between particles at fluid-fluid interfaces (*i.e.*, oil-water and air-water interfaces) scale as *r^−3^* [5,6,7,8,9]. Such abnormally strong and long-ranged repulsive interactions stem from the dipole-dipole interactions due to asymmetric charge distribution across the interface [5,6,7] and/or the presence of the surface residual charges in the apolar phase (oil or air) [8,9]. The power law exponent of the repulsive interaction at the fluid-fluid interface (*U_rep_* ~ *r^−3^*) has also been demonstrated by the direct measurements using the optical laser tweezers [8,18,23,29]. The particles *i* and *j* possess their own potentials, the self-potentials, Ω_i_ and Ω_j_, respectively [31]. Notably, it was previously found that the measured values of self-potentials are heterogeneous, and that the random combination of the self-potentials corresponds to the pair interaction magnitude (*a_ij_* = Ω_i_Ω_j_) and follows a gamma distribution:

(2) $$f\left(a;k,\theta \right)=a^{k-1}\frac{e^{-a/\theta }}{\theta^{k}\Gamma \left(k\right)}$$

where the shape and scale parameters are *k* = 4.36; and *θ* = 5.48 × 10^7^ pNµmR^3^, respectively; and Γ(k) is the gamma function [31]. To introduce the heterogeneity of self-potential to MC simulations, the values of self-potentials for a sufficiently large number of particles (500) are regenerated in the range of the measured self-potentials. Then, a set of self-potentials for *N* particles is randomly selected among the regenerated self-potential values for further simulation studies.

*N* particles tend to collect near the bottom due to gravity (Figure 1). The corresponding potential energy, upon the incorporation of buoyancy, can be expressed as follows:

(3) $$\frac{U_{pot}}{k_{B}T}=g\left(\rho_{p}V_{p}-\rho_{w}V_{pw}-\rho_{o}V_{po}\right)\sum_{k=1}^{N}h_{k}$$

where *V_p_* is the volume of a particle; *ρ* is the density; and *g* is the acceleration due to gravity [30]. The vertical distance between the *k*-th particle and the bottom of the oil lens, *h_k_*, is determined by interpolating the interface height at the radial distance (*λ*) of the particle from the center of the oil lens (Figure 1). The particle volumes immersed in the water and oil phases are given as:

(4) $$V_{pw}=\frac{\pi R^{3}}{3}\left(2+3\cos\theta_{c}-\cos^{3}\theta_{c}\right)$$

in water and

(5) $$V_{po}=\frac{\pi R^{3}}{3}\left(2-3\cos\theta_{c}+\cos^{3}\theta_{c}\right)$$

in oil. The gel-trapping method is used to measure the three-phase contact angle of a particle (*θ_c_* = 105°) at the oil-water interface [30,35].

Assuming pairwise additivity [17], the net interaction energy of particle *i* is the sum of all pair interactions with its surrounding particles *j*; that is:

(6) $$U_{net,i}=U_{pot,i}+\sum_{j,j\neq i}^{N}U_{rep,ij}$$

Notably, the pairwise additivity of the interaction potentials was previously justified when the interactions between the particles are sufficiently strong and long-ranged, such as the electrostatic repulsions between particles at fluid-fluid interfaces [17]. The total energy of an assembly composed of *N* particles at the curved oil-water interface is then given by:

(7) $$U_{tot}=\sum_{i}^{N}U_{net,i}$$

The total energy is used to determine whether to accept or reject a new configuration after particle *i* is randomly moved. Initial positions of the number of particles in MC simulations are randomly generated at the curved oil-water interface. The size of the oil lens does not significantly affect the assembly behaviors [31]; therefore, its radius is arbitrarily chosen to be *R_lens_* = 2 mm.

## 4. Conclusions

The assembly behavior of colloidal particles at the curved oil-water interface has been investigated. Based on the experimental observations and MC simulations, assembly behaviors of several particles at the curved oil-water interface can be summarized as follows. (1) The values of self-potential and its heterogeneity directly affect the assembly behaviors (*i.e.*, the diverse assembly pattern formation); (2) the formation of assembly structure also depends on the assembly kinetics; (3) the presence of the two structures demonstrates that one of the structures is kinetically stable and that there is an essential corresponding energy barrier between the two structures. The two structures can be transformed from one to the other upon the introduction of external stimuli, such as convection or an optical force, which likely imparts the activation energy required to overcome the energy barrier. Notably, this assembly behavior can be typically applied for cases when the particles assemble a maximum two different structures (*N* ≤ 20). Based on preliminary results after performing more MC simulations, more than two different assembly structures can occur for the larger number of particle systems, suggesting the possibility of increasing the number of kinetically stable assembly patterns; and (4) a very interesting feature observed in the assembly patterns is that the particles with low values of self-potentials likely form inner particles and the particles with relatively high values of self-potentials stay outside, such that the resulting structure composed of several particles is energetically favorable. Based on this assembly behavior, it may be possible to sort particles in the order of their self-potential values by subjecting sufficiently strong external energy to the sample such as mechanical vibration and, therefore, the particles that are radially distributed at the curved interface can adopt a thermodynamically stable structure.

In addition, interactions (electrostatic repulsions) between colloidal particles at a fluid-fluid interface do not significantly depend on the interfacial curvature when the curvature radius is sufficiently large in comparison to the particle size; however, the assembly structures and behaviors at a curved interface are notably different from those at a planar interface. For a planar interface, when homogeneous repulsive interaction potentials are employed in the simulations, the resulting assembly structures should show a hexagonal honeycomb pattern with six neighboring particles. In contrast, the introduction of heterogeneous potentials to the simulations results in an assembly which includes colloidal defects with particles possessing five or seven neighboring particles [29]. The assembly structures at a curved interface necessarily possess colloidal defects, regardless of the introduction of either heterogeneous or homogeneous potentials to the simulations. This is analogous to a football, which cannot be organized only with hexagons. Therefore, this work has the potential to provide a fundamental basis from which to understand the intimate relationship between small-scale measurements such as interparticle interactions and assembly behaviors, and the large-scale properties of materials composed of many identical particles such as micro- and macro-structures and interfacial rheology.
